# Effects of Ursolic Acid Derivatives on Caco-2 Cells and Their Alleviating Role in Streptozocin-Induced Type 2 Diabetic Rats

**DOI:** 10.3390/molecules190812559

**Published:** 2014-08-19

**Authors:** Panpan Wu, Ping He, Suqing Zhao, Tianming Huang, Yujing Lu, Kun Zhang

**Affiliations:** Department of Pharmaceutical Engineering, Faculty of Chemical Engineering and Light Industry, Guangdong University of Technology, Guangzhou 510006, China

**Keywords:** diabetes mellitus, ursolic acid derivatives, Caco-2 cell, 2-NBDG, streptozotocin (STZ)-induced diabetic rat

## Abstract

In this study, the effect and mechanism of a series of ursolic acid (UA) derivatives on glucose uptake were investigated in a Caco-2 cells model. Their effect on hyperglycemia, hyperlipidemia and oxidative stress were also demonstrated in streptozocin (STZ)-induced diabetic rats. 2-[N-(7-nitrobenz-2-oxa-1,3-diazol-4-yl)amino]-2-deoxy-glucose (2-NBDG) was used as a fluorescein in Caco-2 cells model to screen UA derivatives by glucose uptake and expression of glucose transporter protein (SGLT-1, GLUT-2). Moreover, STZ-induced diabetic rats were administered with these derivatives for 4 weeks of treatment. The fasting blood glucose (FBG), insulin levels, biochemical parameters, lipid levels, and oxidative stress markers were finally evaluated. The results of this study indicated that compounds **10** and **11** significantly inhibited 2-NBDG uptake under both Na^+^-dependent and Na^+^-independent conditions by decreasing SGLT-1 and GLUT-2 expression in the Caco-2 cells model. Further *in vivo* studies revealed that compound **10** significantly reduced hyperglycemia by increasing levels of serum insulin, total protein, and albumin, while the fasting blood glucose, body weight and food intake were restored much closer to those of normal rats. Compounds **10** and **11** showed hypolipidemic activity by decreasing the total amounts of cholesterol (TC) and triglycerides (TG). Furthermore, compound **10** showed antioxidant potential which was confirmed by elevation of glutathione (GSH) and superoxide dismutase (SOD) and reduction of malondialdehyde (MDA) levels in the liver and kidney of diabetic rats. It was concluded that compound **10** caused an apparent inhibition of intestinal glucose uptake in Caco-2 cells and hypoglycemia, hypolipidemia and augmented oxidative stress in STZ-induced diabetic rats. Thus, compound **10** could be developed as a potentially complementary therapeutic or prophylactic agent for diabetics mellitus and its complications.

## 1. Introduction

Currently, there are more than 347 million people worldwide that have diabetes, making diabetes mellitus (DM) one of the most serious chronic diseases in the world. The WHO projects that diabetes will be the 7th leading cause of death in 2030 [1]. DM is a metabolic disorder characterized by glucose intolerance and changes in protein and lipid metabolism; it has become more and more common because of unhealthy diet and sedentary lifestyle in the general public [2,3]. Long-term diabetic patients who are treated ineffectively suffer from complications such as nephropathy, retinopathy, heart disease, peripheral neuropathy and so on [4]. The risks of acquiring cardiovascular disease, stroke, and cancer are higher in DM patients [5,6,7]. In addition, the overall risk of dying among people with DM linked complications is at least double the risk of their peers without DM [8]. Hypoglycemic agents, including insulin and other modern oral drugs, can control the blood glucose levels, but they may also have limited efficacy and many other undesirable effects. Therefore, it is evident that there is an urgent need for more effective therapeutic agents.

Pentacyclic triterpenes are abundantly distributed in herbal medicine. Ursolic acid (UA, 3β-hydroxy-urs-12-en-28-oic, **1**), which is a pentacyclic triterpene, has been reported to possess many bioactivities, such as antitumor [9,10], anti-inflammatory [11] anti-diabetic activities [12] and so on. The mechanism of its anti-diabetic activity has been proven by alleviating diabetic complications like atherosclerosis and nephropathy [13,14]. However, there are few studies focused on the properties of UA derivatives on glucose transport and streptozotocin (STZ)-induced DM rats. In light of this, we hypothesized that an additional mechanism of action might be implicated in the anti-diabetic activity of UA derivatives. In this study, we implemented a fluorometric method to evaluate a multitude of properties of glucose uptake in Caco-2 cells with 2-NBDG [15,16], which has several advantages over other available glucose tracers [17]. Glucose can be absorbed into blood capillaries when dietary carbohydrates are digested into monosaccharides [18]. Therefore, a strategy of managing diabetes is to inhibit the glucose uptake in the intestines to control the blood glucose level [19]. In the intestines, glucose is transported mainly by two transporters (SGLT-1, GLUT-2), depending on the luminal glucose concentration.

The detrimental effect of diabetic complications is complicated through oxidative stress [20]. Diabetes is usually associated with augmented production of molecules of reactive oxygen species (ROS) or abated antioxidant defense systems, both of which result in enhanced oxidative damage and lead to ROS-mediated diabetic pathogenesis [21]. Compared with healthy adults, type 2 DM patients have lower levels of glutathione (GSH) and superoxide dismutase (SOD), the primary endogenous antioxidants. In contrast, malondialdehyde (MDA), a highly toxic byproduct generated partially by lipid oxidation and ROS, is increased in patients with diabetes [22]. Thus, enhancing the antioxidant capacity is one method to relieve diabetes and its complications.

**Figure 1:**
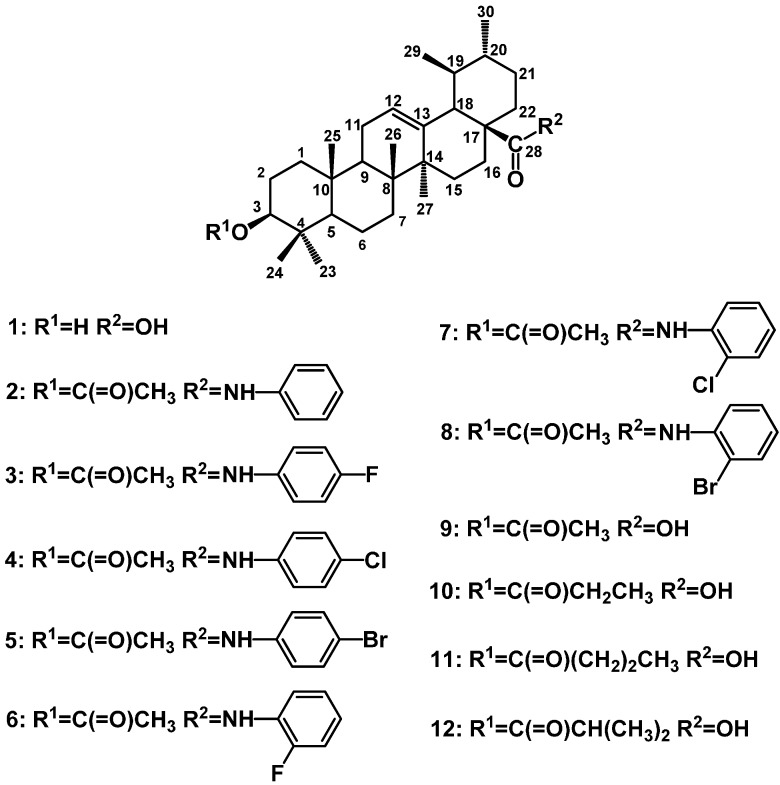
The structure of evaluated compounds **1**–**12**.

In the present study, an investigation was undertaken to evaluate the effects of UA and its derivatives shown in Figure 1 [23], which were synthesized in our laboratory, on the expression of glucose uptake and glucose transporter protein (SGLT-1, GLUT-2) in Caco-2 cells model. Furthermore, the anti-hyperglycemic, anti-hyperlipidemic, anti-oxidant inhibitory effects of these derivatives in STZ-induced DM rats were also investigated.

## 2. Results and Discussion

### 2.1. Cell Toxicity

As shown in Table 1, the cytotoxicity of UA and its derivatives against Caco-2 cells was studied. The results indicate that the viability of Caco-2 cells was greater than 90%, with the exception of cells treated with compound **6**, for which the cell viability was 86.12%, thus, it is not suitable for *in vivo* studies.

**Table 1:** Cell toxicity of **UA** and its derivatives on Caco-2 cells.

Compound	Inhibition Rate %	Compound	Inhibition Rate %
**1**	9.88 ± 6.51	8	9.86 ± 2.68
**2**	5.02 ± 2.38	9	4.40 ± 3.09
**3**	8.65 ± 3.49	10	7.84 ± 4.15
**4**	8.53 ± 5.69	11	2.29 ± 6.08
**5**	8.72 ± 1.18	12	7.67 ± 5.36
**6**	13.88 ± 4.52	Phlorizin	7.70 ± 3.91
**7**	8.14 ± 3.81	Phloretin	4.70 ± 2.59

Values denote mean ± SD, *n* = 4. Inhibitory percentage of cells treated with each compound at a concentration of 100 μM for 24 h.

### 2.2. Effects of **UA** and Its Derivatives on Glucose Uptake by Caco-2 Cells

**Figure 2:**
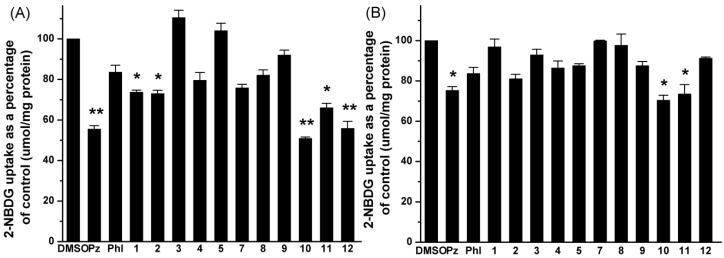
Effects of selected compounds on glucose uptake in Caco-2 cells under condition of (**A**) Na^+^-dependent and (**B**) Na^+^-independent. Cells were treated with vehicle, positive and the indicated compounds (100 μM). The data reported represent the means (*n* = 3) ± SD. * *p* < 0.05, ** *p* < 0.01.

Caco-2 cell are widely used as a model for intestinal absorption studies [24]. As illustrated in the sodium-dependent glucose uptake study (Figure 2A), compounds **1**–**2** and **10**–**12** caused a statistically significant reduction in glucose uptake, especially compounds **10** and **12** (** *p* < 0.01). Phlorizin and phloretin were used as positive controls. The effect of these compounds reducing 2-NBDG adsorption are as follows: phlorizin (56% reduction), compound **1** (74% reduction), compound **2** (73% reduction), compound **10** (51% reduction), compound **11** (66% reduction), compound **12** (56% reduction). The other compounds had no effect on glucose uptake under these conditions.

The sodium-independent glucose uptake study was also performed like the sodium-dependent one, but NaCl and Na_2_HPO_4_ were replaced by KCl and K_2_HPO_4_. As shown in Figure 2B, only phlorizin and compounds **10** and **11** had a significant reduction effect on glucose uptake (* *p* < 0.05), which was significantly decreased to 75%, 70%, 74% of the control value, whereas the other compounds had no detectable effect under these conditions.

### 2.3. Effect of **UA** and Its Derivatives on Glucose Transporter Protein Expression

**Figure 3:**
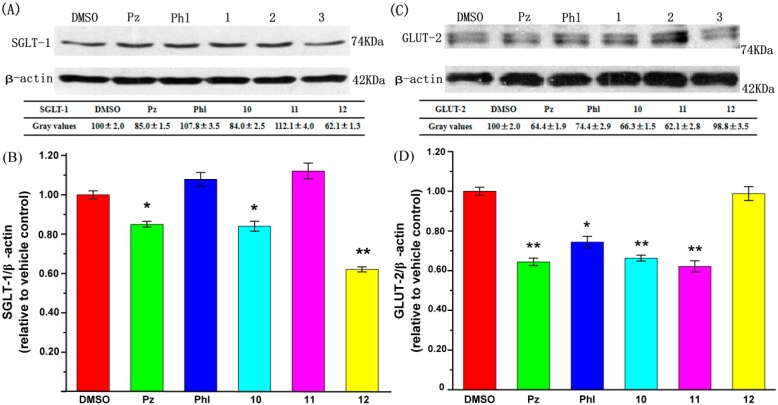
Effects of selected compounds on protein expression level of SGLT-1 and GLUT-2. (**A**) Representive SGLT-1 immunoblots; (**B**) Quantitation of SGLT-1 immunoblots; (**C**) Representive SGLT-2 immunoblots; (**D**) Quantitation of SGLT-2 immunoblots. Protein expression levels of SGLT-1 and GLUT-2 were quantified with respect to the level of β-actin and expressed as relative changes in comparison to the vehicle control. The data reported represent the means (*n* = 3) ± SD. * *p* < 0.05, ** *p* < 0.01.

SGLT-1 and GLUT-2 are the most significant transporters involved in glucose transportation in the intestines [25,26]. Caco-2 cells were treated with 0.1% DMSO, 100 μM phlorizin, 100 μM phloretin, 100 μM compounds **10**–**12** for 6 h, then Caco-2 cells were lysed and analysed by western blotting. The effect of compounds **10**–**12** on glucose uptake suggested that these compounds may exert their effects through modulation of glucose transporter expression. As shown in Figure 3, compounds **10** and **12** are able to significantly reduce the protein expression of SGLT-1, particularly compound **12**, whereas compounds **10** and **11** had significant effects on reducing the expression of protein GLUT-2.

### 2.4. Acute Oral Toxicity Study of **UA** and Its Derivatives

The acute oral toxicity study revealed these tested compounds’ non-toxic nature; no toxic reactions or lethality were observed at the doses of 100, 200 and 500 mg/kg. Based on the result, 100 mg/kg was selected as the maximum dose for oral administration.

### 2.5. Effects of **UA** and Its Derivatives on Fasting Blood Glucose Levels

During the 4 week treatment, the blood glucose levels were changed in normal, diabetic control, and diabetic treated rats. The results are presented in Table 2. The fasting blood glucose levels of normal rats were not changed until the end of the period, while the blood glucose were significantly increased in untreated diabetic rats as compared with normal control. However, the high levels of blood glucose decreased in diabetic rats treated with compound **10** and glibenclamide. At the end of the 4 week treatment, 100 mg/kg of compound **10** decreased blood glucose levels (49.6%) as compared with diabetic control. The glibenclamide group achieved 54.4% drop in blood glucose levels. In addition, compounds **1**, **11** and **12** had no significant effect on lowing fasting blood glucose levels.

**Table 2:** Effects of glibenclamide and the selected compounds on fasting blood glucose levels of normal, diabetic control, and diabetic treated rats.

Group	Fasting Blood Glucose Level (mg/dL)				
	Week 0	Week 1	Week 2	Week 3	Week 4
Normal control	73.80 ± 4.50	75.60 ± 7.20	72.00 ± 6.30	72.00 ± 5.40	81.00 ± 2.16
Diabetic control	320.40 ± 20.52	401.40 ± 22.50	428.40 ± 50.40	433.80 ± 39.06	446.40 ± 31.68
Glibenclamide (10 mg/kg)	338.40 ± 16.38	271.80 ± 18.36 *	183.60 ± 11.70 *	216.00 ± 9.72 *	203.40 ± 13.50 *
Compound **1** (100 mg/kg)	342.00 ± 19.08	405.00 ± 17.46	392.40 ± 13.32	379.80 ± 19.44	396.00 ± 16.92
Compound **10** (100 mg/kg)	320.40 ± 12.96	270.00 ± 16.74 *	286.20 ± 18.90	234.00 ± 14.58 *	225.00 ± 11.70 *
Compound **11** (100 mg/kg)	318.60 ± 17.64	365.40 ± 20.34	352.80 ± 27.36	324.00 ± 23.04	351.00 ± 26.64
Compound **12** (100 mg/kg)	325.80 ± 14.58	392.40 ± 36.54	379.80 ± 30.60	408.60 ± 20.52	432.00 ± 28.26

The data reported represent the means (*n* = 6) ± SD; * Mean values that are significantly different from diabetic control group (*p* < 0.05).

### 2.6. Effect of **UA** and Its Derivatives on Serum Biochemical Parameters

**Figure 4:**
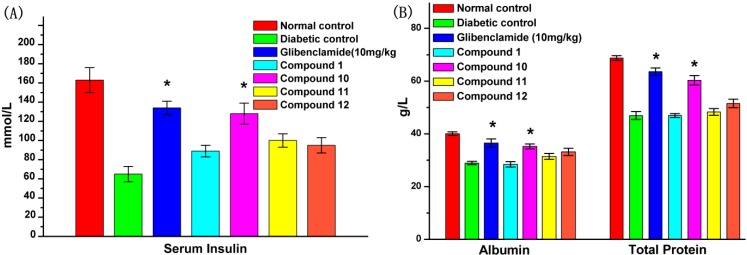
Effects of compounds **1**, **10**, **11** and **12** on serum biochemical parameters of STZ-induced diabetic rats in comparison with normal and diabetic control rats after 4 weeks treatment. At the end of the treatment period, rats were fasted for 12 h and blood was drawn to collect the serum. Panels denote (**A**) serum insulin; (**B**) albumin and total protein levels. The data reported represent the means (*n* = 6) ± SD. * Mean values that are significantly different from diabetic control group (*p* < 0.05).

After 4 weeks of treatment, the serum insulin, total protein and albumin levels in untreated diabetic rats were significantly reduced compared to the normal control group. Figure 4 shows that after 4 weeks of administration of glibenclamide (10 mg/kg) and compound **10** (100 mg/kg), the serum insulin, total protein and albumin levels in diabetic rats were increased significantly as compared with the diabetic control. Compounds **1**, **11** and **12** had a certain but non-significant effect of increasing insulin levels. In addition, compound **1**, **11** and **12** caused no appreciable improvement in increasing total protein and albumin levels in diabetic rats.

### 2.7. Effect of **UA** and Its Derivatives on Body Weight and Food Intake

**Figure 5:**
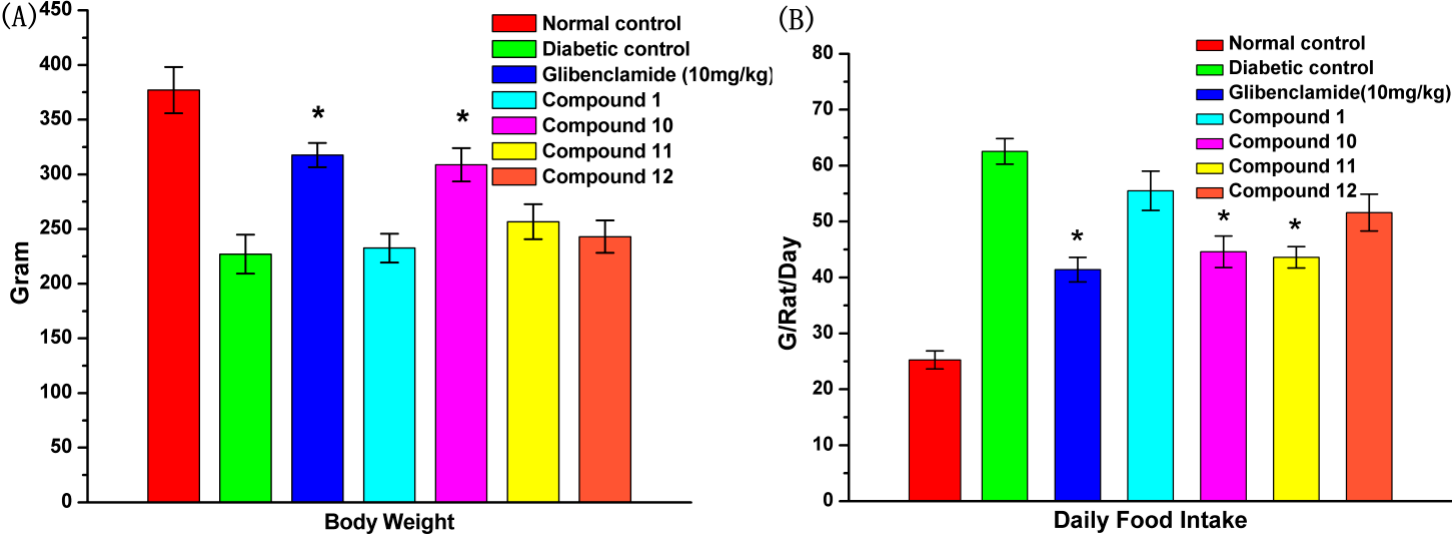
Effects of compounds **1**, **10**, **11** and **12** on (**A**) body weight and (**B**) daily food intake of STZ-induced diabetic rats in comparison with normal and diabetic control rats. The data reported represent the means (*n* = 6) ± SD. * Significant difference compared to diabetic control (*p* < 0.05).

The effect of tested compounds **1**, **10**, **11** and **12** on changes of body weight and daily food intake are presented in Figure 5. As shown in Figure 5A, it could be concluded that there was a significant increase in body weight for compound **10-**treated diabetic rats when compared with the diabetic control and the glibenclamide treatment group by the end of the treatment period, while the other compounds had no such appreciable effect. Moreover, as shown in Figure 5B, there was a significant increase in food intake in diabetic rats. After oral administration of glibenclamide, compound **10** and **11**, the three compounds could reduce daily food intake significantly compared with the untreated diabetic rats. In contrast, compounds **1** and **12** presented no obvious decrease.

### 2.8. Effect of **UA** and Its Derivatives on Hyperlipidemia

**Figure 6:**
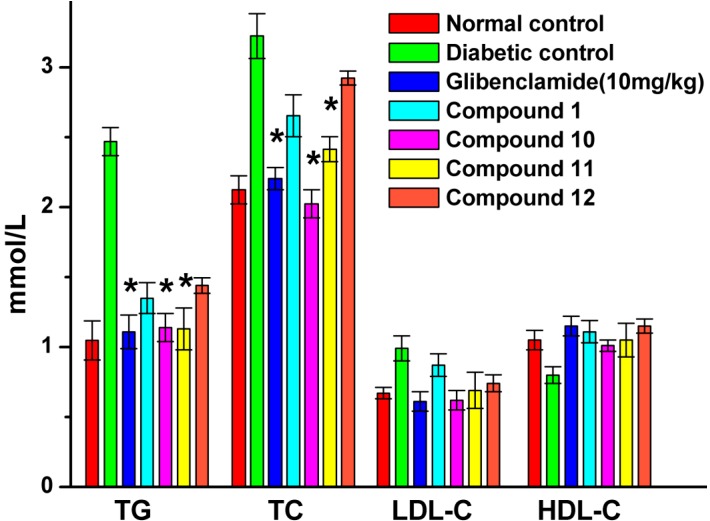
Effects of compounds **1**, **10**, **11** and **12** on serum lipid profiles of STZ-induced diabetic rats in comparison with normal and diabetic control rats after 4 weeks of treatment. At the end of the treatment period, rats were fasted for 12 h and blood was drawn to collect the serum. The data reported represent the means (*n* = 6) ± SD. * Significant difference compared to diabetic control (*p* < 0.05).

Figure 6 shows the effect of glibenclamide and UA derivatives on serum TG, TC, LDL-C and HDL-C levels in diabetic rats and normal control.

Serum TG, TC and LDL-C levels were significantly elevated in diabetic control when compared with the normal control, while the HDL-C levels in diabetic control were significantly decreased when compared with those in normal control. However, Serum TC, TG levels were significantly decreased after treatment with glibenclamide, compounds **10** and **11**. Compared with diabetic control, serum LDL-C levels were also lowered, while HDL-C levels were higher with the tested compound treatment but none of gave a significant effect (*p* < 0.05).

### 2.9. Effect of **UA** and Its Derivatives on Oxidative Stress

**Figure 7:**
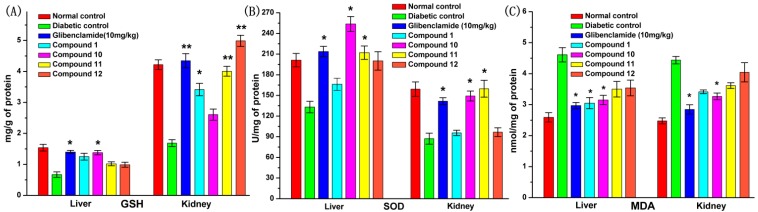
Antioxidant effect of compounds **1**, **10**, **11** and **12** on the kidney and liver of STZ-induced diabetic rats. Panels denote (**A**) GSH, (**B**) SOD and (**C**) MDA levels. The data reported represent the means (*n* = 6) ± SD. * Significant difference compared to diabetic control (*p* < 0.05), ** Significant very difference compared to diabetic control (*p* < 0.01).

Figure 7 reveals that the levels of GSH and SOD in diabetic control were reduced while the levels of MDA were significantly increased when compared with those in normal control. All tested compounds produced an increase in GSH levels in liver and kidney. In particularly glibenclamide and compound **10** demonstrated significant effects on the liver, while compounds **1**, **11** and **12** showed a great effect on kidney. There was a great improvement of SOD level in the liver and kidney of diabetic rats after treatment with glibenclamide, compounds **10** and **11**. Compared with the diabetic control, the MDA levels in the liver of diabetic rats were obviously down-regulated by daily administration of glibenclamide, compounds **1** and **10**, while the MDA levels were significantly reduced by glibenclamide and compound **10** (*p* < 0.05). 

### 2.10. Discussion

UA has been reported to possess a wide range of bioactivities, including positive effects in curing various complications of diabetes and lowing blood glucose levels [13,27,28]. However, the anti-diabetic potential of UA derivatives and their mechanism(s) of action have not been thoroughly investigated. In the present study, the activities of UA and some of its derivatives were assayed in a Caco-2 cell model. Moreover, the potential of these derivatives in STZ-induced diabetic rats model was also investigated.

The results of MTT assays indicated that at a concentration of 100 μM none of the derivatives showed toxicity to Caco-2 cells, except for compound **6**, which is in line with the results of acute oral toxicity studies *in vivo*. The effects of these derivatives on glucose uptake under Na^+^-dependent and Na^+^-independent conditions in Caco-2 cells were assessed. The fluorescent glucose analog probe 2-NBDG was used to measure glucose uptake rates [29,30,31]. Specificity of glucose transporters to the analog probe was confirmed by inhibition of uptake of 2-NBDG by D-glucose and phlorizin [13,32].

Before a meal, glucose concentration in plasma is much higher than that in lumen [33]. Any glucose could be quickly captured by SGLT-1, because SGLT-1 is a low-capacity, high-affinity transporter and is the only transporter capable of moving glucose against a concentration gradient; GLUT-2 is a high-capacity, low-affinity facilitative transporter that equilibrates glucose between plasma and enterocytes [34,35]. To gain insight into the effects of UA and its derivatives on glucose uptake, we studied the most important glucose transporter proteins as the major transporters are responsible for the absorption of glucose by Caco-2 cells. Under sodium-dependent conditions, it is expected that both SGLT-1 and GLUT-2 would operated at the apical surface to absorb glucose, while under sodium-free conditions, only GLUT-2 worked to carry out glucose uptake [36]. As shown in Figure 2 and Figure 3, compound **10** could reduce the glucose uptake by the reduction of both SGLT-1 and GLUT-2 expression under Na^+^-dependent conditions. The effect of compound **11** relied on the decrease of GLUT-2, while compound **12** relied on the inhibition of the expression of SGLT-1. The results also suggested that the inhibition of GLUT-2 by the tested compounds was greater than that of SGLT-1 [37]. Phlorizin, compounds **10** and **11** all reduced glucose uptake under both Na^+^-dependent and Na^+^-free conditions, which meant that the inhibition of GLUT-2 by these three compounds was greater than that of SGLT-1. This was consistent with the results of a protein expression study.

According to the result of an *in vitro* study, the anti-diabetic potential of UA and its derivatives was investigated in STZ-induced DM rats. After daily administration of the tested compounds, blood glucose levels were significantly reduced by compound **10**, whereas insulin levels were increased, and compound **10** (100 mg/kg) had a similar effect as glibenclamide (10 mg/kg) (Figure 5). Elevated insulin levels in diabetics usually normalize the serum and tissue proteins by improving protein synthesis and reducing protein degradation or protein glycosylation [38]. The characteristic loss of body weight associated with STZ-induced DM rats could be attributed to dehydration and catabolism of fat or breakdown of tissue proteins, which leads to muscle waste [29]. The recovery of body weight observed in the compound **10** treated DM rats could be the result of increased glucose uptake, insulin secretion and decreased fasting blood glucose levels, as an indication of improved glycemic control in the rats.

The dyslipidemia associated with diabetes is typically a combination of hyperlipidemia with insulin resistance and even modest abdominal obesity involves elevation of LDL cholesterol, an increase of TG and a decrease of HDL cholesterol [39]. The DM rats treated with compounds **10** and **11** showed that the two compounds have significantly effect in reducing serum TC and TG levels (Figure 6), which were demonstrated as the effectiveness of these compounds against experimental STZ-induced DM in rats.

Streptozotocin has been widely used to induce DM, which may result from an increase of reactive oxygen species (ROS) and the inhibition of free radicals defense system [40]. ROS may cause peroxidation by react with lipids, resulting in elevated lipid peroxidation. The increase of lipid peroxidation might be an indication of a decrease of enzymatic and non-enzymatic antioxidants in defense mechanisms [41]. It is well known that glutathione (GSH) is a major endogenous antioxidant, one of its major functions being that its sulfhydryl (SH) group is a strong nucleophile that confers antioxidant protection [42], so the depletion of liver and kidney GSH levels reflect augmented oxidative stress [43,44]. Compound **10** showed a significant restoration of GSH content in the liver of DM rats (Figure 7A). The natural cellular antioxidant enzyme superoxide dismutase (SOD) plays a pivotal role in oxygen defense metabolism by reducing superoxide to water and molecular oxygen [45,46]. Compounds **10** and **11** showed a significant increase in SOD levels in the liver and kidney in DM rats model (Figure 7B). Malondialdehyde (MDA) as the final product of lipid peroxidation is an indicator of peroxidation level. That is, the higher the concentration of the MDA, the more serious peroxide levels in the body are. Compound **10** showed a significant elevation of MDA levels in the liver and kidney in DM rats (Figure 7C). After treatment with compound **10**, there was an increase of the activities of SOD and GSH, and a decrease in the activity of MDA. These results indicated that compound **10** could effectively protect cells against oxidative stress by scavenging free radicals.

Hyperlipidemia is characterized by the metabolic syndrome in addition to a disproportionate elevation of apo B levels. The measurement of fasting glucose and apo B in addition to the fasting lipid profile can help to estimate the risk of coronary artery disease and to guide treatment decisions in patients with the metabolic syndrome [47]. It is known that there are a certain number of physiological antioxidants in the type 2 DM model. The depletion of antioxidants in the diabetic condition is a main cause of diabetic pathogenesis. Present therapeutic strategies typically attempt to relieve the clinical manifestation of diabetes and its complications, which justifies the therapeutic use of anti-diabetic agents coupled with antioxidants in cases of fast emergence of diabetes [48].

In this study, the bioactivity of UA and its derivatives in Caco-2 cells model and STZ-induced diabetic rats were studied. Regarding the mechanism of action of compound **10** could be concluded that it played an important role in both Na^+^-dependent and Na^+^-independent conditions by inhibiting glucose transporter protein expression, and compound **10** has significant capability of reducing hyperglycemia, hyperlipidemia and oxidative stress in STZ-induced DM rats, so compound **10** not only can reduce the absorption of glucose in STZ-induced DM rats intestinal, but also has the effect of alleviating diabetic complications and may therefore contribute to effective diabetes management in the future.

## 3. Experimental Section

### 3.1. Preparation of **UA** and Its Derivatives

UA was purchased from Nanjing Zelang Medical Technology Co., Ltd. (Nanjing, China), with over 98% purity. UA derivatives (see Figure 1) were obtained from our research team [23]. These compounds were purified by column chromatography and their structures were all confirmed.

### 3.2. In vitro Cell Culture

Human intestinal Caco-2 cells (ATCC, Rockville, MD, USA) were incubated at 37 °C in a humidified atmosphere of 5% CO_2_ in air. The cells were cultured in Dulbecco’s modified Eagle’s modified Eagle’s medium (DMEM) with 10% heat-inactivated fetal bovine serum, 1% non-essential amino acids, 1% L-glutamine, 1% penicillin/streptomycin (Gibco Life Technologies, Grand Island, NY, USA). The cells were sub-cultured at confluence by 0.05% trypsin-0.5 mM EDTA treatment before they were applied in the experiment.

### 3.3. Cytotoxicity Assay

A MTT test was conducted to determine the possible toxicity of UA and its derivatives to Caco-2 cells. Briefly, cells were seeded at a density of 1 × 10^4^ cells/mL in a 96-well plate and incubated for 24 h. The next day, cells were incubated with vehicle (0.1% DMSO) and compounds at 100, 50, 25, 12.5 μmol/L for 24 h, and then 5 g/L MTT solution (20 μL) was added for 4 h. Absorbance at 570 nm was measured using a Multimodel Plate Reader (Infinite 200, Tecan, Mannedorf, Switzerland). The results were expressed as the percentage of control cells demonstrating cell viability after treated with the testing compounds.

### 3.4. Glucose Uptake by Caco-2 Cell Monolayers

Caco-2 cells, which were derived from human colon adenocarcinoma, were selected for intestinal absorption studies because these cells express the morphological characteristics and most of the functional properties of differentiated small-intestinal enterocytes [49]. Caco-2 cells were seeded on 24-well plates at the density of 2 × 10^5^ cells/well. The medium was changed every 1–2 days, and the culture was carried out for 13 days. Then Caco-2 cells were placed in serum-free media for 24 h and then they were washed twice with Hanks’ balanced salt solution (HBSS, pH 7.5, 140 mM NaCl, 5 mM KCl, 1.2 mM Na_2_HPO_4_, 2 mM CaCl_2_, 1.2 mM MgSO_4_, 20 mM HEPES, 0.2% bovine serum albumin) before the uptake studies. When a sodium free buffer was required, NaCl and Na_2_HPO_4_ were replaced with equal amounts of KCl and K_2_HPO_4_, respectively. After washing, the cells were incubated for 15 min at room temperature in HBSS before the commencement of this experiment. The uptake studies were initiated by adding HBSS containing either control or test solution (100 μM) and 2-BNDG (100 μM) for 30 min at 37 °C. Glucose uptake was stopped by adding twofold volume of ice-cold PBS and the wells were washed with ice-cold PBS three times. Fluorescent intensity was measured by a Multimodel Plate Reader (Tecan Infinity 200) with the 485 nm_ex_ and 535 nm_emiss_ filter set before and after adding 2-NBDG. Then the cells were lysed in 200 μL lysis buffer (10 mM Tris-HCl pH = 7.4, 150 mM NaCl, 1% Triton-x-100, 1 mM EDTA, 0.1% SDS) and supplemented immediately before they were processed with 10 μg/mL PMSF. Cells were allowed to lyse on ice, and then vortexed and sonicated. The lysate was used for protein determination, protein concentrations were determined as described by BCA kit (Boster, Wuhan, China) with bovine serum albumin as standard.

### 3.5. Western Blot Analysis

Caco-2 cells were cultured on 6-well plates at a density of 1 × 10^6^ cells/well for 13 days. The cells were placed in serum-free medium for 24 h prior to the treatment. Then UA and its derivatives (100 μM) were added, after 6 h incubation, the cells were immediately washed three times with ice-cold PBS and lysed in 200 μL lysis buffer. Cells were allowed to lyse on ice, and then scraped, vortexed, sonicated and stored at −80 °C for further analysis. Protein concentrations were determined as described and cell lysate was subjected to western blot analysis [49]. Briefly, proteins with same amount were denatured in sample buffer, separated on 8% SDS-PAGE gel, and transferred onto a polyvinylidene diflouride membrane. The membranes were blocked for 1 h at 37 °C with 10% defatted milk in Tris-buffered saline (10 mM Tris-HCl, pH 7.5 and 137 mM NaCl) containing 0.05% Tween 20 (TBST). Membranes were then incubated overnight at 4 °C in blocking buffer with antibodies for GLUT-2, SGLT-1 and β-actin (EMD, Millipore, Billerica, MA, USA). Membranes were washed with TBST and incubated 1 h with appropriate secondary antibodies. Revelation was performed by using the enhanced chemiluminescence reagent and blue-light sensitive film. Densitometric analysis of films was performed with a Hewlett-Packard scanner equipped with a transparent adaptor and UN-SCAN-IT software.

### 3.6. In Vivo Experimental Animals

Adult male albino rats of Sprague-Dawley rats (180–220 g) were obtained from the Guangdong Medical Lab Animal Center, and maintained under standard laboratory conditions in a temperature (25–30 °C) and light-controlled (12-h light/dark cycle) room with 35%–60% humidity. The animals were acclimatized for 10 days before the experiments and provided with rodent chow and water *ad libitum*. The investigation was conducted in accordance with the Guide for the Care and Use of Laboratory Animals and approved by the institutional Animal Ethics Committee.

### 3.7. Oral Acute Toxicity Studies

Healthy adult SD rats were used for this test. The rats were fasted overnight and divided into 13 groups (*n* = 4), the rats were orally fed with compound **1**, **10**, **11** and **12** with an increasing dose of 100, 200 and 500 mg/kg body weight [50,51]. Control group was given vehicle solution (0.5% CMC-Na). These rats were continuously observed for 2 h for their behavioral, neurological, and autonomic symptoms and then observed once more after a period of 24 to 72 h for any sign of lethality or death [52].

### 3.8. Induction of Diabetes

Diabetes was induced by a single intraperitoneal injection of streptozotocin (Sigma-Aldrich, St. Louis, MO, USA) at a dose of 65 mg/kg body weight. STZ was dissolved in 0.1 M cold citrate buffer, pH 4.5. Control group were injected with citrate buffer alone. After 7 days for the development of diabetes, plasma glucose levels were determined of each rat. Rats with fasting blood glucose (FBG) range of above 16.7 mmol/L were considered as DM rats and were used for further study [53].

### 3.9. Experimental Procedure

According to the body weight and plasma glucose levels the rats were divided randomly into seven groups with six rats each and treated as follows:

Group I: Normal rats treated with 0.5% CMC-Na (2 mL/200 g body weight).
Group II: Diabetic rats treated with 0.5% CMC-Na.
Group III: Diabetic rats treated with 10 mg/kg of glibenclamide.
Group IV: Diabetic rats treated with 100 mg/kg of compound **1** (UA).
Group V: Diabetic rats treated with 100 mg/kg of compound **10**.
Group VI: Diabetic rats treated with 100 mg/kg of compound **11**.
Group VII: Diabetic rats treated with 100 mg/kg of compound **12**.

The treatment was continued daily for 28 days. Blood glucose levels and body weight were measured every week to ascertain the status of diabetes. After 4 weeks of treatment, the animals were fasted for 12 h and sacrificed by cervical dislocation under mild anaesthetization, blood was collected into heparinized tubes and centrifuged at 2,000 rpm for 10 min. Livers and kidneys were removed, washed and homogenized in ice-cold normal saline. This homogenates were centrifuged at 3,000 rpm for 10 min at 4 °C, and the supernatants were collected for the estimate of antioxidant markers.

### 3.10. Biochemical Analysis

Fasting blood glucose levels were estimated on days 0, 7, 14, 28 with the single touch glucometer (Life Scan, Johnson & Johnson Company, New Brunswick, NJ, USA). Serum insulin level was measured by use of a commercial immunoassay kit by enzyme linked immunosorbent assay (Uscn. Life Science Inc., Wuhan, China). Serum total protein, albumin, triglycerides (TG), total cholesterol (TC), high-density lipoprotein cholesterol (HDL-C), and low-density lipoprotein cholesterol (LDL-C) were measured with an automatic biochemical analyzer (Sinnowa D240, Nanjing, China).

### 3.11. Assessment of Oxidative Stress Markers

The tissue homogenates of liver and kidney were used for the estimate of antioxidant markers. Glutathione (GSH) level was measured with 5, 5'-dithiobis-(2-nitrobenzoic acid) (DTNB) as described by Sedlak and Lindsay [54]. Superoxide dismutase (SOD) activity was assessed according to the method of Sinha [55]. Malondialdehyde (MDA) activity was estimated with the thiobarbituric acid method [56].

### 3.12. Statistical Analysis

All experiment results were expressed with mean ± standard deviation (SD). The significant differences between the means of the experimental groups were determined with the analysis of variance (ANOVA), followed by a Tukey–Kramer multiple comparisons test (Graph Pad version 5.0; Graph Pad Software Inc., San Diego, CA, USA). The values were considered significant when *p* < 0.05.

## 4. Conclusions

In summary, our study suggests that compound **10** displays an inhibitory effect on 2-NBDG uptake through inhibiting SGLT-1 and GLUT-2 transporter protein expression in Caco-2 cells. This observation was corroborated by its benefits in attenuating hyperglycemia, hyperlipidemia and oxidative stress in a DM rat model. Moreover, compound **10** is easily obtained from UA by one step structure modification [23]. These finding are supportive to the application of compound **10** as a potential source for the discovery of active new and active oral medicine for future therapy of diabetes and its complications.

## Abbreviations

2-NBDG: 2-[N-(7-nitrobenz-2-oxa-1, 3-diazol-4-yl) amino]-2-deoxyglucose
SGLT-1: Na^+^-dependent glucose transporter
GLUT-2: Na^+^-independent glucose transporter
DM: diabetes mellitus
UA: ursolic acid
STZ: streptozotocin
ROS: reactive oxygen species
GSH: glutathione
SOD: superoxide dismutase
MDA: malondialdehyde
TG: triglyceride
TC: total cholesterol
LDL-C: low density lipoprotein cholesterol
HDL-C: high density lipoprotein cholesterol

## References

[1] WHO “Fact Sheet # 312” to Be Found under. http://www.who.int/mediacentre/factsheets/fs312/en/.

[2] Tuomilehto J., Lindström J., Eriksson J.G., Valle T.T., Hämäläinen H., Ilanne-Parikka P., Keinänen-Kiukaanniemi S., Laakso M., Louheranta A., Rastas M. (2001). Prevention of type 2 diabetes mellitus by changes in lifestyle among subjects with impaired glucose tolerance. N. Engl. J. Med..

[3] Zimmet P., Alberti K.G., Shaw J. (2001). Global and societal implications of the diabetes epidemic. Nature.

[4] Lopez-Candales A. (2001). Metabolic syndrome X: A comprehensive review of the pathophysiology and recommended therapy. J. Med..

[5] Hays N.P., Galassetti P.R., Coker R.H. (2008). Prevention and treatment of type 2 diabetes: Current role of lifestyle, natural product, and pharmacological interventions. Pharmacol. Ther..

[6] Hung H.Y., Qian K., Morris-Natschke S.L., Hsu C.S., Lee K.H. (2012). Recent discovery of plant-derived anti-diabetic natural products. Nat. Prod. Rep..

[7] Jia W., Gao W., Tang L. (2003). Antidiabetic herbal drugs officially approved in China. Phytother. Res..

[8] Roglic G., Unwin N., Bennett P.H., Mathers C., Tuomilehto J., Nag S., Connolly V., King H. (2005). The burden of mortality attributable to diabetes: Realistic estimates for the year 2000. Diabetes Care.

[9] Harmand P.O., Duval R., Delage C., Simon A. (2005). Ursolic acid induces apoptosis through mitochondrial intrinsic pathway and caspase-3 activation in M4Beu melanoma cells. Int. J. Cancer.

[10] Kim D.K., Baek J.H., Kang C.M., Yoo M., Sung J.W., Kim D.K., Chung H.Y., Kim N.D., Choi Y.H., Lee S.H. (2000). Apoptotic activity of ursolic acid may correlate with the inhibition of initiation of DNA replication. Int. J. Cancer.

[11] Baricevic D., Sosa S., Della L.R., Tubaro A., Simonovska B., Krasna A., Zupancic A. (2001). Topical anti-inflammatory activity of Salvia officinalis L. leaves: The relevance of ursolic acid. J. Ethnopharmacol..

[12] Kazmi I., Rahman M., Afzal M., Gupta G., Saleem S., Afzal O., Shaharyar M.A., Nautiyal U., Ahmed S., Anwar F. (2012). Anti-diabetic potential of ursolic acid stearoyl glucoside: A new triterpenic gycosidic ester from Lantana camara. Fitoterapia.

[13] Ullevig S.L., Zhao Q., Zamora D., Asmis R. (2011). Ursolic acid protects diabetic mice against monocyte dysfunction and accelerated atherosclerosis. Atherosclerosis.

[14] Zhou Y., Li J.S., Zhang X., Wu Y.J., Huang K., Zheng L. (2010). Ursolic acid inhibits early lesions of diabetic nephropathy. Int. J. Mol. Med..

[15] Zou C., Wang Y., Shen Z. (2005). 2-NBDG as a fluorescent indicator for direct glucose uptake measurement. J. Biochem. Biophys. Methods.

[16] Yamada K., Saito M., Matsuoka H., Inagaki N. (2007). A real-time method of imaging glucose uptake in single, living mammalian cells. Nat. Protoc..

[17] Hassanein M., Weidow B., Koehler E., Bakane N., Garbett S., Shyr Y., Quaranta V. (2011). Development of high-throughput quantitative assays for glucose uptake in cancer cell lines. Mol. Imaging Biol..

[18] Nistor B.L., Martineau L.C., Benhaddou-Andaloussi A., Arnason J.T., Levy E., Haddad P.S. (2010). Inhibition of intestinal glucose absorption by anti-diabetic medicinal plants derived from the James Bay Cree traditional pharmacopeia. J. Ethnopharmacol..

[19] Kim H.K., Baek S.S., Cho H.Y. (2011). Inhibitory effect of pomegranate on intestinal sodium dependent glucose uptake. Am. J. Chin. Med..

[20] Haidara M.A., Yassin H.Z., Rateb M., Ammar H., Zorkani M.A. (2006). Role of oxidative stress in development of cardiovascular complications in diabetes mellitus. Curr. Vasc. Pharmacol..

[21] Pitozzi V., Giovannelli L., Bardini G., Rotella C.M., Dolara P. (2003). Oxidative DNA damage in peripheral blood cells in type 2 diabetes mellitus: Higher vulnerability of polymorphonuclear leukocytes. Mutat. Res..

[22] Evans J.L. (2007). Antioxidants: Do they have a role in the treatment of insulin resistance?. Indian J. Med. Res..

[23] Wu P.P., Zhang K., Lu Y.J., He P., Zhao S.Q. (2014). *In vitro* and *in vivo* evaluation of the antidiabetic activity of ursolic acid derivatives. Eur. J. Med. Chem..

[24] Sambuy Y., de Angelis I., Ranaldi G., Scarino M.L., Stammati A., Zucco F. (2005). The Caco-2 cell line as a model of the intestinal barrier: Influence of cell and culture-related factors on Caco-2 cell functional characteristics. Cell. Biol. Toxicol..

[25] Delie F., Rubas W. (1997). A human colonic cell line sharing similarities with enterocytes as a model to examine oral absorption: advantages and limitations of the Caco-2 model. Drug Carrier Syst..

[26] Mahraoui L., Rodolosse A., Barbat A., Dussaulx E., Zweibaum A., Rousset M., Brot-Laroche E. (1994). Presence and differential expression of SGLT1, GLUT1, GLUT2, GLUT3 and GLUT5 hexose-transporter mRNAs in Caco-2 cell clones in relation to cell growth and glucose consumption. Biochem. J..

[27] Jang S.M., Yee S.T., Choi J., Choi M.S., Do G.M., Jeon S.M., Yeo J., Kim M.J., Seo K.I., Lee M.K. (2009). Ursolic acid enhances the cellular immune system and pancreatic beta-cell function in streptozotocin-induced diabetic mice fed a high-fat diet. Int. Immunopharmacol..

[28] Jang S.M., Kim M.J., Choi M.S., Kwon E.Y., Lee M.K. (2010). Inhibitory effects of ursolic acid on hepatic polyol pathway and glucose production in streptozotocin-induced diabetic mice. Metabolism.

[29] Arya A., Looi C.Y., Cheah S.C., Mustafa M.R., Mohd M.A. (2012). Anti-diabetic effects of Centratherum anthelminticum seeds methanolic fraction on pancreatic cells, beta-TC6 and its alleviating role in type 2 diabetic rats. J. Ethnopharmacol..

[30] Poitout V., Stout L.E., Armstrong M.B., Walseth T.F., Sorenson R.L., Robertson R.P. (1995). Morphological and functional characterization of beta TC-6 cells-an insulin-secreting cell line derived from transgenic mice. Diabetes.

[31] Yamada K., Nakata M., Horimoto N., Saito M., Matsuoka H., Inagaki N. (2000). Measurement of glucose uptake and intracellular calcium concentration in single, living pancreatic beta-cells. J. Biol. Chem..

[32] Masumoto S., Akimoto Y., Oike H., Kobori M. (2009). Dietary phloridzin reduces blood glucose levels and reverses Sglt1 expression in the small intestine in streptozotocin-induced diabetic mice. J. Agric. Food. Chem..

[33] Goto T., Horita M., Nagai H., Nagatomo A., Nishida N., Matsuura Y., Nagaoka S. (2012). Tiliroside, a glycosidic flavonoid, inhibits carbohydrate digestion and glucose absorption in the gastrointestinal tract. Mol. Nutr. Food Res..

[34] Kellett G.L., Brot-Laroche E., Mace O.J., Leturque A. (2008). Sugar absorption in the intestine: The role of GLUT2. A. Leturque. Annu. Rev. Nutr..

[35] Brown G.K. (2000). Glucose transporters: Structure, function and consequences of deficiency. Metab. Dis..

[36] Manzano S., Williamson G. (2010). Polyphenols and phenolic acids from strawberry and apple decrease glucose uptake and transport by human intestinal Caco-2 cells. Mol. Nutr. Food Res..

[37] Kellett G.L., Brot-Laroche E. (2005). Apical GLUT2: A major pathway of intestinal sugar absorption. Diabetes.

[38] Almdal T.P., Vilstrup H. (1988). Strict insulin therapy normalises organ nitrogen contents and the capacity of urea nitrogen synthesis in experimental diabetes in rats. Diabetologia.

[39] Knopp R.H., Retzlaff B., Aikawa K., Kahn S.E. (2003). Management of patients with diabetic hyperlipidemia. Am. J. Cardiol..

[40] Kavalali G., Tuncel H., Goksel S., Hatemi H.H. (2003). Hypoglycemic activity of Urtica pilulifera in streptozotocin-diabetic rats. J. Ethnopharmacol..

[41] Cemek M., Kaga S., Simsek N., Buyukokuroglu M.E., Konuk M. (2008). Antihyperglycemic and antioxidative potential of Matricaria chamomilla L. in streptozotocin-induced diabetic rats. J. Nat. Med..

[42] Fang Y.Z., Yang S., Wu G. (2002). Free radicals, antioxidants, and nutrition. Nutrition.

[43] Griesmacher A., Kindhauser M., Andert S.E., Schreiner W., Toma C., Knoebl P., Pietschmann P., Prager R., Schnack C., Schernthaner G. (1995). Enhanced serum levels of thiobarbituric-acid-reactive substances in diabetes mellitus. Am. J. Med..

[44] Dewanjee S., Das A.K., Sahu R., Gangopadhyay M. (2009). Antidiabetic activity of Diospyros peregrina fruit: effect on hyperglycemia, hyperlipidemia and augmented oxidative stress in experimental type 2 diabetes. Food Chem. Toxicol..

[45] Blake D.R., Allen R.E., Lunec J. (1987). Free radicals in biological systems-A review orientated to inflammatory processes. Br. Med. Bull..

[46] Lin Y.F., Tsai H.L., Lee Y.C., Chang S.J. (2005). Maternal vitamin E supplementation affects the antioxidant capability and oxidative status of hatching chicks. J. Nutr..

[47] Carr M.C., Brunzell J.D. (2004). Abdominal obesity and dyslipidemia in the metabolic syndrome: Importance of type 2 diabetes and familial combined hyperlipidemia in coronary artery disease risk. J. Clin. Endocrinol. Metab..

[48] Yoshihara T., Kumashiro N., Kanazawa Y., Mita T., Sakurai Y., Kawai J., Abe M., Motojima K., Hara K., Yamazaki Y. (2006). Therapeutic efficacy of mitiglinide combined with once daily insulin glargine after switching from multiple daily insulin regimen of aspart insulin and glargine in patients with type 2 diabetes mellitus. Endocr. J..

[49] Burnette W.N. (1981). “Western blotting”: Electrophoretic transfer of proteins from sodium dodecyl sulfate-Polyacrylamide gels to unmodified nitrocellulose and radiographic detection with antibody and radioiodinated protein A. Anal. Biochem..

[50] Shirwaikar A., Rajendran K., Barik R. (2006). Effect of aqueous bark extract of Garuga pinnata Roxb. in streptozotocin-nicotinamide induced type-II diabetes mellitus. J. Ethnopharmacol..

[51] Ghosh M.N. (2007). Fundamentals of experimental pharmacology. Indian J. Pharmacol..

[52] Turner R.A., Hebborn P. (1965). Screening Methods in Pharmacology.

[53] Sarkar S., Pranava M., Marita R. (1996). Demonstration of the hypoglycemic action of Momordica charantia in a validated animal model of diabetes. Pharmacol. Res..

[54] Sedlak J., Lindsay R.H. (1968). Estimation of total, protein-bound, and nonprotein sulfhydryl groups in tissue with Ellman’s reagent. Anal. Biochem..

[55] Sinha A.K. (1972). Colorimetric assay of catalase. Anal. Biochem..

[56] Draper H.H., Hadley M. (1990). Malondialdehyde determination as index of lipid peroxidation. Method. Enzymol..

